# Profil épidémio-clinique des atteintes dermatologiques chez le noir Africain en hémodialyse chronique

**DOI:** 10.11604/pamj.2016.25.142.7193

**Published:** 2016-11-11

**Authors:** Emmanuel Armand Kouotou, François Kaze Folefack, Joël Tameyi Tatsa, Isidore Sieleunou, Jobert Richie Nansseu Njingang, Gloria Ashuntantang, Anne-Cécile Zoung-Kanyi Bissek

**Affiliations:** 1 Centre Hospitalier et Universitaire de Yaoundé, Yaoundé, Cameroun; 2 Hôpital Général de Yaoundé, Yaoundé, Cameroun; 3 Département de Médecine Interne et Spécialités, Faculté de Médecine et des Sciences Biomédicales, Université de Yaoundé I, Yaoundé, Cameroun; 4 School of Public Health, University of Montréal, Montréal, Canada; 5 Unité de Prise en Charge de la Drépanocytose, Centre Mère et Enfant de la Fondation Chantal Biya, Yaoundé, Cameroun

**Keywords:** Atteintes dermatologiques, hémodialyse, noir africain, Yaoundé, Cameroun, Skin damages, hemodialysis, black african, Yaounde, Cameroon

## Abstract

**Introduction:**

Les manifestations dermatologiques sont fréquentes chez les hémodialysés chroniques et traduiraient une atteinte systémique. Notre objectif était de déterminer le profil épidémio-clinique sur peau noire à Yaoundé au Cameroun.

**Méthodes:**

Il s’agissait d’une étude transversale, menée de Février à Mai 2014 dans les deux centres d’hémodialyse de Yaoundé, incluant tout hémodialysé chronique depuis au moins 3 mois, et chez qui étaient conduits un interrogatoire et un examen dermatologique. Les tests de Chi carré et ***t*** de Student (ou équivalents) ont été utilisés pour l’analyse statistique, avec le seuil de signification fixé à p<0,05.

**Résultats:**

Au total, 112 patients (dont 78 (69,9%) hommes) d’un âge moyen de 48,6 ± 13 ans et une durée moyenne en dialyse de 46,3 ± 37 mois étaient recrutés. Les lésions dermatologiques étaient présentes chez 94 (83,9%) patients. La xérose cutanée (63,3%), le prurit (37,5%), la mélanodermie (34,8%), l’acné (12,5%) et les ongles équi-segmentés (10,7%) étaient les principales manifestations dermatologiques. La xérose était associée à l’anurie (p=0,0001) et à l’âge avancé (p=0,032) ; la mélanodermie à l’anurie (p=0,042) et à l’ancienneté en dialyse (p=0,027) alors que les ongles équi-segmentés étaient associés au jeune âge (p=0,018) et aux dialyses bihebdomadaires (p=0,01).

**Conclusion:**

Les atteintes dermatologiques sont fréquentes et dominées par la xérose, le prurit et la mélanodermie chez l’hémodialysé chronique à Yaoundé. La sous dialyse, l’âge avancé, l’anurie et l’ancienneté en dialyse étaient les facteurs associés.

## Abstract (en)

**Introduction:**

Dermatologic manifestations are common among patients on chronic hemodialysis and may represent systemic involvement. Our study aims to determine the epidemiological and clinical profile of skin damages in black patients living in Yaounde, Cameroon.

**Methods:**

We conducted a cross sectional study including all patients receiving chronic haemodialysis treatment for at least 3 months in two hemodialysis centers in Yaounde from February to May 2014. Patients underwent an interview and a dermatological examination. Chi-squared tests and Student’s t-test (or equivalents) were used for statistical analysis, with significance level at p <0.05.

**Results:**

A total of 112 patients (78 (69.9%) men) with an average age of 48.6 ± 13 years and a mean duration of dialysis of 46,3 ± 37 months were included in the study. Skin lesions were present in 94 (83.9%) patients. Xerosis (63.3%), pruritus (37.5%), melanoderma (34.8%), acne (12.5%) and half and half nails (10.7%) were the most common dermatologic manifestations. Xerosis was associated with anuria (p = 0.0001) and advanced age (p = 0.032); melanoderma was associated with anuria (p = 0.042) and time spent on dialysis (p = 0.027) while half and half nails were associated with young age (p = 0.018) and biweekly dialysis (p = 0.01 ).

**Conclusion:**

Skin damages are frequent and dominated by xerosis, pruritus and melanoderma in patients on chronic hemodialysis living in Yaounde. Biweekly dialysis, advanced age, anuria and time spent on dialysis were associated factors.

## Introduction

La progression de la maladie rénale chronique (MRC) s’accompagne de complications multi-systémiques parmi lesquelles l’atteinte dermatologique (AD). On estime que 50 à 100% des patients en insuffisance rénale chronique terminale présentent au moins une complication dermatologique [1, 2]. Bah et al [3] ont montré que la plupart des AD apparaissent à partir d’une clairance de la créatinine inférieure à 30 ml/min, et s’intensifient avec la dégradation de la fonction rénale vers le stade terminal. Ces AD sont le plus souvent bénignes et non spécifiques, mais présentent parfois des degrés de gravité variables, allant du simple inconfort à une complication sévère [2]. En Afrique, Falodun et al. [4] au Nigeria avaient retrouvé une prévalence des AD de 89% chez les patients hémodialysés chroniques; elles étaient dominées par la xérose cutanée, le prurit et les troubles des phanères qui pouvaient être des indicateurs cliniques de chronicité de l’insuffisance rénale dans les pays à revenus limités [3]. En raison de la référence tardive des patients insuffisants rénaux aux néphrologues [5, 6] et de l’accroissement du nombre d’hémodialysés chroniques au Cameroun [7], la symptomatologie cutanée pourrait fonctionner comme une fenêtre pour l’évaluation et l’appréciation de la fonction rénale ainsi que de l’efficacité de la dialyse. C’est dans ce contexte que nous nous sommes proposés de mener cette étude afin de déterminer le profil épidémio-clinque des manifestations dermatologiques rencontrées chez les patients hémodialysés chroniques dans les centres de dialyse de la ville de Yaoundé au Cameroun.

## Méthodes

Il s’agissait d’une étude transversale, descriptive et analytique qui s’est déroulée de Février à Mai 2014 dans les centres de dialyse du Centre Hospitalier et Universitaire de Yaoundé (CHUY) et de l’Hôpital Général de Yaoundé (HGY). Pour réaliser cette étude, une clairance éthique a été obtenue auprès du comité d’éthique de la Faculté de Médecine et des Sciences Biomédicales de l’Université de Yaoundé I, Cameroun.

Nous avons inclus consécutivement tout patient hémodialysé chronique depuis au moins trois mois ayant signé le formulaire de consentement éclairé. Les patients hémodialysés chroniques en vacances ou en transit dans les centres étaient exclus de l’étude. Les données sociodémographiques, cliniques et biologiques de chaque patient, collectées à partir d’un questionnaire préétabli, étaient obtenues à partir de l’interrogatoire, du dossier médical et du dossier de dialyse. Les données biologiques utilisées dataient de moins de trois mois. L’examen dermatologique complet était réalisé par le dermatologue à la fin de la séance de dialyse chez tout participant à l’étude, complètement dévêtu, avec ou sans plaintes particulières. L’interrogatoire permettait de rechercher la présence d’une symptomatologie dermatologique.

Les données ont été saisies et les analyses statistiques réalisées dans le logiciel Epi-Info TM version 7.1.3 (Centre for Disease Control, Atlanta, USA). Les variables quantitatives ont été décrites par leurs moyennes et écart-types et les variables qualitatives, par leurs effectifs et pourcentages. La comparaison entre les moyennes a été déterminée à l’aide du test t de Student. Pour l’analyse des variances nous avons utilisé les tests ANOVA et de Kruskall-wallis. Les comparaisons entre variables qualitatives ont été faites à l’aide du test de Khi-2. Les valeurs de probabilité p < 0,05 ont été considérées comme statistiquement significatives.

## Résultats

### Caractéristiques de la population étudiée

Sur les 196 patients rencontrés dans les centres de dialyse de la ville de Yaoundé pendant toute la durée de l’étude, 112 patients (57,1%) ont répondu aux critères d’inclusion parmi lesquels 58 (51,8%) provenaient de l’HGY et 54 (48,2%) du CHUY. Comme présenté dans le Tableau 1, il y avait une prédominance masculine avec un sexe ratio de 2,3. L’âge moyen était de 48,6 ± 13 ans avec des extrêmes de 16 à 82 ans. La durée moyenne en dialyse était de 46,3 ± 37 mois (extrêmes : 3-174 mois). Quatre-vingt-quatre patients (75%) patients étaient anuriques, et seulement 36 patients (32,1%) pratiquaient des dialyses trihebdomadaires. Le sexe masculin était associé à une moyenne d’âge élevée (p=0,001) et une prévalence élevée d’anurie (p=0,0003) et du diabète (p=0,033).

**Tableau 1:** Caractéristiques générales de la population en fonction du sexe

Caractéristiques	Sexe		Total	p
	Féminin n (%)	Masculin n (%)		
**n (%)**	34 (30,3)	78 (69,7)	112 (100)	
Age moyen (années ± ET)	43 ± 15	51 ± 12	49 ± 13	**0,002**
Durée en dialyse (mois ± ET)	41 ± 36	40 ± 34	40 ± 34	0,88
**Diurèse résiduelle**				
Persistante	16 (47)	12(15)	28(25,0)	**0,0004**
Absente	18 (53)	66(85)	84 (75,0)	
**Fréquence de la dialyse**				
Bihebdomadaire	24(71)	52(67)	76 (67,9)	0,68
Trihebdomadaire	10(29)	26(33)	36 (32,1)	
**Comorbidités**				
HTA	18(52,9)	56(71,8)	74(66,1)	0,085
Diabète	4(11,8)	25(32,1)	29(25,9)	0,033
Hépatite C	8(23,5)	22(28,2)	30(26,8)	0,60

### Atteintes dermatologiques rencontrées

**Figure 1:**
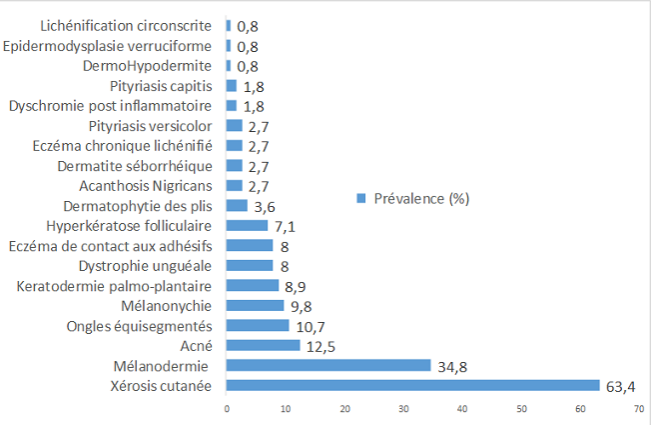
Principales atteintes dermatologiques présentés par nos patients hémodialysés

**Figure 2:**
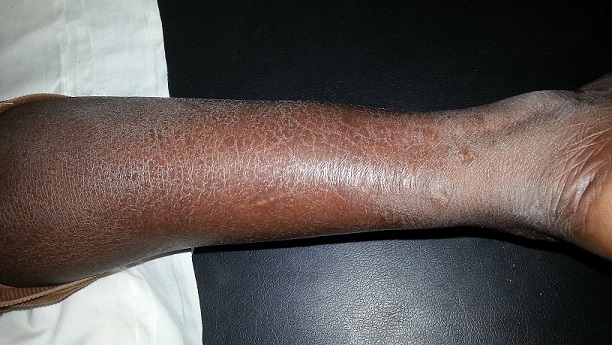
Xérose ichtyosiforme de la jambe

Dans notre série, 94 patients (83,9%) présentaient au moins une manifestation dermatologique. Le prurit était le principal signe fonctionnel et relevé chez 42 patients (37,5%). La xérose cutanée (63,4%) et la mélanodermie (34,8%) étaient les principales lésions dermatologiques observées (Figure 1). La xérose cutanée réalisait dans notre cohorte une forme sévère ichtyosiforme chez 29 patients (40,8%) (Figure 2). Elle était observée sur une peau atrophique chez 17 patients (soit 89,5% des patients ayant une atrophie cutanée). Quant à la mélanodermie, elle prédominait sur les zones photo-exposées.

### Facteurs associés aux principales atteintes dermatologiques

**Figure 3:**
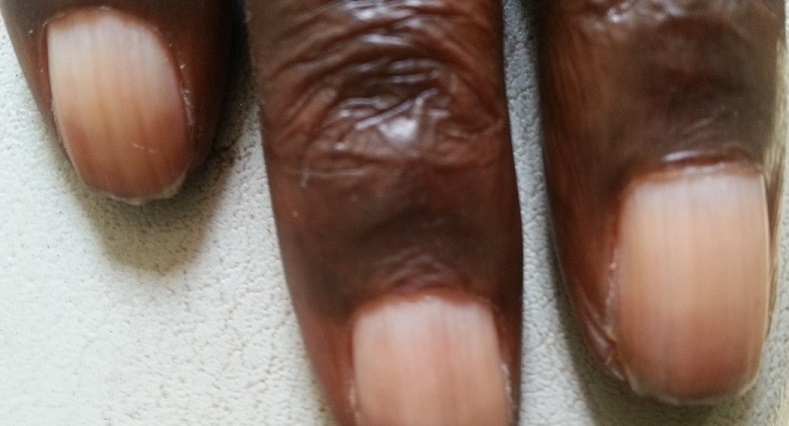
Ongles équi-segmentés (hyperpigmentation distale et zone claire proximale)

A l’analyse du Tableau 2, La xérose cutanée était associée à l’anurie (p=0,0001) et à l’âge avancé (p=0,032), alors que la mélanodermie pour sa part était associée à l’anurie (p=0,042) et à l’ancienneté en dialyse (p=0,027). Cependant, les ongles équi-segmentés (Figure 3) étaient associés au jeune âge (p=0,018) et aux dialyses bihebdomadaires (p=0,01).

**Tableau 2:** Caractéristique des patients hémodialysés présentant une xérose cutanée, des ongles équi-segmengtés ou la mélanodermie

Caractéristiques	Xérose cutanée						Ongles equi-segmentés			Mélanodermie		
	Non	Oui			Valeur p		Oui	Non	Valeur p	Oui	Non	Valeur p
		Xérose Légère à modérée	Xérose Ichtyosiforme	Total	Présence/ Absence de xérose	Intensité xérose						
**Effectifs (%)**	41(36,6)	42(37,5)	29(25,9)	71(63,4)			12(10,7)	100(89,3)		39(34,8)	73(75,2)	
Age moyen en année ± ET [Médiane en années]	45±14 [43]	50±11 [50]	53±11 [54]	51 ± 13 [52]	0,012	0,032	40±11 [37]	49±13 [52]	0,018	48±14 [50]	50±14 [51]	0,47
Durée en dialyse en mois ± ET [Médiane en mois]	34±33 [19]	43±35 [39]	44±35 [48]	44±35 [45]	0,15	0,36	39±31 [31]	40±35 [29]	0,91	49±32 [51]	34±34 [20]	0,027
**Sexe**					0,45	0,08			0,52			0,87
Masculin	26(63,4)	27(64,3)	25(86,2)	52(73,2)			8(66,7)	70(70,0)		27(69,2)	51(69,9)	
Féminin	15(36,6)	15(35,7)	4(3,8)	19(26,8)			4(33,3)	30(30,0)		12(30,8)	22(30,1)	
**Diurèse résiduelle**					0,01	0,0001						0,042
Persistante	19(46,3)	8(19,0)	1(3,4)	9(12,7)			4(50,0)	24(24,0)	0,48	9(23,1)	34(46,6)	
Absente	22(53,7)	34(81,0)	28(96,6)	62(87,3)			8(50,0)	76(76,0)		30(76,9)	39(53,4)	
**Fréquence de dialyse**												
Bihebdomadaire	26(63,4)	27(64,3)	23(79,3)	50(70,4)	0,09	0,30	12(100,0)	64(64,0)	0,01	28(71,8)	48(65,7)	0,88
Trihebdomadaire	15(36,6)	15(35,7)	6(20,7)	21(29,6)			0(0,0)	36(36,0)		11(29,2)	25(34,3)	

## Discussion

Nous avons réalisé une étude transversale descriptive et analytique pour laquelle 112 patients hémodialysés ont été colligés dans les deux centres de dialyse de la ville de Yaoundé au Cameroun. Il s’agissait d’un échantillon d’adultes jeunes, à majorité masculine similaire aux résultats de la littérature en Afrique sub-saharienne [5, 8, 9]. Ce profil de patients pourrait être lié aux facteurs socio-économiques et socio-environnementaux parmi lesquels l’insuffisance de soins primaires [10], et à la composante génétique qui serait à l’origine d’une progression rapide de la MRC [11, 12]. On estime que la MRC est plus fréquente en Afrique subsaharienne que dans le reste du monde, et que sa vitesse de progression vers le stade terminal y est plus élevée [13].

Nous avons retrouvé une forte prévalence des lésions dermatologiques (83,9%), proche des valeurs retrouvées dans les séries africaines et occidentales [1, 4, 14, 15]. Cette forte prévalence pourrait s’expliquer par l’âge avancé d’une bonne partie de nos patients, par le caractère photosensibilisant de certains produits utilisés, les comorbidités relevées et même par le caractère racial (sujet noir africain). La principale plainte dermatologique était le prurit, proche des 44,5% retrouvés par Masmoudi et al. au Maroc [14]. Cette prévalence restait cependant inférieure aux trouvailles européennes et américaines qui situent la prévalence du prurit respectivement entre 50% et 90% au cours de la MRC terminale [2, 16], et supérieure aux résultats de Falodoun et al. au Nigeria en 2011 [4], et Dahbi et al. au Maroc en 2012 [17] qui ont rapporté une prévalence du prurit chez les patients dialysés respectivement de 27,6% et de 20,7%. Ces observations pourraient s’expliquer par l’amélioration des techniques de dialyse.

Quant à la xérose cutanée, nos résultats se rapprochent de ceux de Falodoun et al. au Nigeria [4] et de Masmoudi et al. au Maroc [14] qui ont observé des prévalences respectives de 69,7% et de 69,0%. Par ailleurs, Dahbi et al. [17] avaient retrouvé que 96,0% des patients hémodialysés présentaient une xérose cutanée. Dans notre série, cette xérose cutanée était associée à l’âge avancé des patients et à l’anurie. Ces résultats se rapportent à l’étio-pathogénie de la xérose cutanée au cours de la MRC. En effet, les auteurs évoquent un certain nombre de perturbations biologiques et biochimiques dépendant de la fonction rénale résiduelle parmi lesquelles la diminution de la teneur en eau de l’épiderme, l’atrophie des glandes sébacées et l’accumulation de molécules telles que la vitamine A [1, 2, 15]. L’atrophie des glandes sébacées semble être liée au vieillissement de la peau qui s’accompagne d’une altération du profil lipidique et d’une diminution de la production de filagrine, à l’origine d’une atrophie cutanée et d’une xérose sénile physiologique [18].

Une mélanodermie était relevée chez 34,8% de nos patients se trouvant dans l’intervalle de la prévalence retrouvée dans la littérature : entre 20 et 70% [1, 2]. Au Nigeria, Falodoun et al [4] retrouvaient une prévalence de 9,2% et Masmoudi et al. au Maroc [14] retrouvaient une mélanodermie chez 17% des patients. Ces différences pourraient s’expliquer par le fait que l’évaluation de la mélanodermie dans ces études comme dans la nôtre était visuelle, basée sur la plainte du patient. Par ailleurs, dans notre série la mélanodermie était plus marquée dans les zones photo-exposées chez nos patients. Ceci pourrait être lié à la présence au niveau de la peau d’une substance photosensible mal dialysable ou peu dialysée et liée au degré d’altération de la fonction rénale. Ceci pourrait être lié à la présence au niveau de la peau d’une substance photosensible mal dialysable ou peu dialysée et liée au degré d’altération de la fonction rénale. En effet, notre étude montre que la mélanodermie était associée à l’anurie et l’ancienneté en dialyse, similaires aux résultats de Lai et al. en 2005 en Taiwan [19].

Les lésions d’acné étaient présentes chez 12,5% de nos patients. La survenue d’acné a été peu rapportée au cours de l’insuffisance rénale chronique terminale. Kolla et al. [20] rapportent une prévalence de 9,2% des lésions acnéiformes dans leur série. Dans une série de 4 cas, Grange et al. [21] relèvent les particularités de l’acné chez les patients dialysés. Ils présentent des lésions réalisant des formes d’acné grave généralement prurigineuses qui surviennent chez des personnes âgées sans antécédents d’acné au préalable.

La proportion de patient présentant des ongles équi-segmentés ou ongles de Lindsay dans notre série (10,7%) était plus faible que celle retrouvée dans la littérature. Les auteurs estiment que 20 à 40% des patients en insuffisance rénale chronique terminale présentent des ongles équi-segmentés [1, 2, 15]. La faible prévalence dans notre série pourrait s’expliquer par la taille réduite de notre échantillon.

Malheureusement, la nature transversale de cette étude, ajoutée à la taille relativement faible de l’échantillon sus-incriminée ne nous ont pas permis de clairement investiguer les facteurs pouvant être associés à l’occurrence d’AD chez nos patients en hémodialyse chronique. Le mode de recrutement non aléatoire pourrait constituer un frein à une généralisation de nos résultats à la population d’hémodialysés chroniques du Cameroun.

## Conclusion

Cette étude montre que les atteintes dermatologiques sont fréquentes chez le noir africain en hémodialyse chronique. La xérose cutanée, le prurit, les troubles de la pigmentation, et les anomalies des phanères et annexes représentent les principales atteintes dermatologiques chez ce groupe de patients. L’âge avancé, l’anurie, l’ancienneté en dialyse et la sous-dialyse constituent des facteurs associés à leur survenue. Ces trouvailles suggèrent tout l’intérêt des consultations dermatologiques lors de la prise en charge des patients suivis pour une maladie rénale chronique.

### Etat des connaissances actuelles sur le sujet

- Un nombre important de patients hémodialysés chroniques font face à des affections dermatologiques plus ou moins invalidantes. Les données actuelles suggèrent une altération cutanée induite par la maladie rénale chronique et potentialisée par les techniques d’hémodialyse;
- La plupart des lésions retrouvées sont variées et contextuelles. Le prurit, affection la plus fréquemment rencontrée, est associé à une altération de la qualité de vie et présente un défi thérapeutique majeur.

### Contribution de notre étude à la connaissance

- Les affections dermatologiques sont fréquentes chez le patient hémodialysé chronique au Cameroun, représentées par la xerose cutanée, le prurit, les troubles de la pigmentation et les anomalies des phanères et annexes;
- L’âge avancé, l’anurie, l’ancienneté en dialyse et la sous-dialyse constituent des facteurs favorisant leur survenue;
- Les efforts pour préserver la diurèse résiduelle chez les patients hémodialysés chroniques pourraient améliorer les symptômes cutanés ressentis.
