# Erratum: Profil épidémio-clinique des chez le noir africain en hémodialyse chronique

**DOI:** 10.11604/pamj.2017.27.134.13052

**Published:** 2017-06-21

**Authors:** The Pan African Medical Journal

**Affiliations:** 1 The Pan African Medical Journal

**Keywords:** Erratum, dermatology, hémodialyse chronique

## Abstract

This erratum corrects “Profil épidémio-clinique des atteintes dermatologiques chez le noir africain en hémodialyse chronique”. The Pan African Medical Journal. 2017;25:142. doi:10.11604/pamj.2016.25.142.7193.

## Erratum

The name of the author of this article [**1**] was misabbreviated on PubMed as “Njingang JR”. The error was not present in both the PDF and HTML versions of the article. The author’s name should be abbreviated as Nansseu JR.
